# Analysis of Putative Epigenetic Regulatory Elements in the *FXN* Genomic Locus

**DOI:** 10.3390/ijms21103410

**Published:** 2020-05-12

**Authors:** Iván Fernández-Frías, Sara Pérez-Luz, Javier Díaz-Nido

**Affiliations:** 1 Departamento Biología Molecular and Centro de Biología Molecular “Severo Ochoa” (UAM-CSIC), Universidad Autónoma de Madrid, 28049 Madrid, Spain; ivan.fernandez@cbm.csic.es (I.F.-F.); javier.diaznido@uam.es (J.D.-N.); 2 Instituto Investigación Sanitaria Puerta de Hierro-Majadahonda, 28222 Madrid, Spain

**Keywords:** frataxin, Friedreich’s ataxia, histone modification, epigenetic regulation and BACs

## Abstract

Friedreich’s ataxia (FRDA) is an autosomal recessive disease caused by an abnormally expanded Guanine-Adenine-Adenine (GAA) repeat sequence within the first intron of the frataxin gene *(FXN*). The molecular mechanisms associated with FRDA are still poorly understood and most studies on *FXN* gene regulation have been focused on the region around the minimal promoter and the region in which triplet expansion occurs. Nevertheless, since there could be more epigenetic changes involved in the reduced levels of *FXN* transcripts, the aim of this study was to obtain a more detailed view of the possible regulatory elements by analyzing data from ENCODE and Roadmap consortia databases. This bioinformatic analysis indicated new putative regulatory regions within the *FXN* genomic locus, including exons, introns, and upstream and downstream regions. Moreover, the region next to the end of intron 4 is of special interest, since the enhancer signals in FRDA-affected tissues are weak or absent in this region, whilst they are strong in the rest of the analyzed tissues. Therefore, these results suggest that there could be a direct relationship between the absence of enhancer sequences in this specific region and their predisposition to be affected in this pathology.

## 1. Background

Friedreich’s ataxia (FRDA, [OMIM229300]), the most common form of hereditary ataxia among individuals of Indo-European origin, is an autosomal recessive disease clinically characterized by progressive cerebellum and spinal cord neurodegeneration, hypertrophic cardiomyopathy, and predisposition to diabetes [1,2]. The disease has a prevalence of approximately 1 in 50,000 people in European populations [3], with an early onset between 10 and 15 years, and most patients normally become wheelchair users around 15 years after the beginning of the symptoms [4]. 

FRDA, firstly described by physician Nicolas Friedreich in 1863, is caused by mutations in the *FXN* gene [MIM 606829], which encodes for a protein called frataxin. The genetic defect associated with the disease is mostly an abnormally expanded GAA repeat sequence within the first intron of the gene [5]. Most FRDA patients (approximately 95%) are homozygous for the expansion and only the remaining 5% are compound heterozygous for the expansion in one allele and generally a point mutation in the other. It has been described that normal alleles contain 8–65 triplets, whereas patient’s alleles typically contain up to 1700 repeats, with a direct correlation between the number of repeats and the severity of the disease [6]. 

At the molecular level, the final consequence of GAA expansion is that FRDA patient cells show a severe deficiency in frataxin transcription [5]; this ultimately leads to a shortage of frataxin, which is a protein that plays an important role in Fe-S cluster biogenesis and in mitochondrial iron metabolism [7]. Frataxin is a mainly mitochondrial protein encoded by a nuclear gene located in the long arm of chromosome 9 (9q21.11), which undergoes two proteolytic cleavages upon entry into the mitochondria [8]. Although at low levels, the mature protein is ubiquitously expressed in healthy individuals, being slightly higher in the dorsal root ganglia, the cerebellum granular layer and tissues with great metabolic demand, such as in the heart and liver [4]. On the other hand, it should be noted that frataxin overexpression is cytotoxic, and thus it requires a tight control of its expression [9]. In fact, it has been proved that iron depletion causes a reduction of frataxin mRNA levels in both control and FRDA-derived patient cells, presumably indicating a negative feedback mechanism between disease phenotype and protein expression [10]. The mature protein is translated from the main transcript (FXN I), although two minority transcripts produced by the alternative splicing of exon 4 have also been described [11], containing exon 5b instead of exon 5a, with or without noncoding exon 6 [5]; however, few data support their implication in the disease. Recently, two new isoforms of the protein have been described, with both lacking a mitochondrial signal peptide and subsequently located either in the cytosol (FXN II) or in the nucleus (FXN III) [12].

## 2. Frataxin Gene Regulation

**Figure 1:**
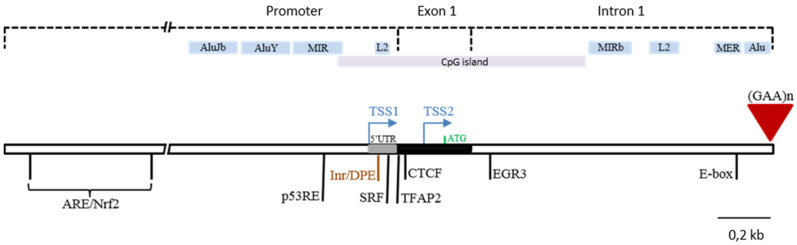
Regulatory elements in the *FXN* locus. Repetitive sequences, regulatory signals, and transcription factor binding sites present in the promoter region, exon 1 and the region surrounding GAA expansion within intron 1. ARE (Antioxidant Response Element); SINE (Short Interspersed Nuclear Element) family, which includes AluJb, AluY, Alu1, MIR and MIRb elements; MER1 (primate-specific MEdium Reiteration 1); Inr (mammalian Initiator); DPE (Downstream Promoter Element); L2, a specific LINE (Long Interspersed Nuclear Element), E-box (Enhancer-box); p53RE (p53 Responsive Element) and CpG (Cytosine-phosphoguanine) island. Transcription factor binding sites Nfr2 (or NFE2L2, Nuclear Factor (Erythroid-derived 2)-Like 2), SRF (Serum Response Factor), TFAP2 (Transcription Factor AP-2) and EGR3 (Early Growth Response factor 3). TSS1 and TSS2 (Transcription Start Site 1 and 2), 5′UTR (Untranslated Region 5′) and GAA triplet expansion ((GAA)n).

The interest in unraveling the molecular mechanisms associated with FRDA has led to advances in certain aspects related to the regulation of the frataxin gene, although this information is still quite incomplete. Two transcription start sites (TSS) are currently described in the *FXN* gene (Figure 1): the first one (TSS1) is located at 221 base pairs upstream from the ATG [5], and the second TSS (TSS2) is located at 62 bp upstream from the ATG (Figure 1); to date, it is still unknown which is the dominant TSS [13]. 

The promoter region also has certain peculiarities. The sequence between 1034 bp upstream and 100 bp downstream from TSS1 plays the main role in the regulation of *FXN*, being considered as the minimal promoter. Although this region lacks TATA-box, it contains an Inr (mammalian Initiator) 24 bp downstream from TSS1 and a Downstream Promoter Element (DPE) located 26 bp downstream from TSS1 (Figure 1). These Inr/DPE elements are considered core promoter regulatory elements, although previous studies indicate that they are not necessary for frataxin expression [14]. 

Despite the fact that frataxin is an essential gene, its promoter is not well-conserved in mammals, mainly due to the insertion of retro-elements, which have been thought to be “evolutionary junk” until recently [15]. However, there are already numerous studies suggesting that these elements could play an important role in the regulation of nearby genes [16]. MIR (Mammalian-wide Interspersed Repeat), AluJb and AluY elements, which belong to the SINE (Short Interspersed Nuclear Element) family, have been described in the promoter, as well as in a region 132 bases downstream from TSS1 that shares a sequence with an L2 element, which is a type of LINE (Long Interspersed Nuclear Element) (Figure 1). Although the exact functions of these elements are still unclear, their deletion has been observed to significantly affect frataxin expression [14].

In the region between the start of intron 1 and the site where the expansion occurs, three repetitive elements are intercalated: a MIRb element, a MER1 (primate-specific MEdium Reiteration 1) element, and part of an Alu element, which is where the expansion of GAA repeats derives from [17] (Figure 1).

Regarding the transcription factors that regulate the expression of *FXN*, there are few data available to date. Previous studies have identified a Serum Response Factor binding site (SRF, 91–110 bp downstream from TSS1), which is important in neuronal development, an AP2 transcription factor binding site (TFAP2; 139–154 bp downstream from TSS1), involved in the development of the neural crest, and an Early Growth Response factor 3 binding site (EGR3; 233–249 bp downstream from TSS2) [18]. The deletion of these sequences significantly reduces the transcription of the gene, although the binding of these transcription factors has not been confirmed [18]. The existence of an E-box (Enhancer-box) is crucial to *FXN* expression and it binds to the MyoD and c-myc transcription factors [17]. A CCCTC binding factor (CTCF) has been described between 154 and 173 bp downstream from TSS1 [19], which is a chromatin insulator protein that prevents the spread of heterochromatin [20]. In addition, there is a p53RE (p53 Responsive Element) 200 bp upstream from TSS1 (Figure 1), indicating that the transcription of *FXN* is activated by p53 [21]. Finally, three important Antioxidant Response Elements (AREs) at 4.9, 5.6, and 16.7 kb upstream from TSS1 have also been described (Figure 1). These ARE elements direct the expression of multiple anti-inflammatory genes, and are related to the presence of transcription factor Nfr2 (or NFE2L2: Nuclear Factor (Erythroid-derived 2) -Like 2) [22], also shown to mediate in anti-inflammatory processes [23,24].

## 3. Deciphering Regulatory Elements in the *FXN* Locus

A scientific field that has acquired special relevance in recent years is the study of epigenetics, which is an essential mechanism in genome regulation [25]. Although the DNA sequence plays an essential role in the regulation of gene expression, epigenetic mechanisms are crucial for their modulation [26]. DNA is wrapped around eight proteins called histones (H2A, H2B, H3, and H4), forming a structure known as nucleosome, the basic unit of chromatin [27]. Gene expression greatly depends on the accessibility of chromatin, which dynamically switches between two states: euchromatin, (the loose/relaxed form of chromatin), which may be active or inactive, and heterochromatin, defined as the condensed and silenced state of chromatin. Post-translational modifications (PTMs) of histones such as phosphorylation, methylation, acetylation, and ubiquitination [28] will affect nucleotide sequence reading [29]. 

In FRDA patients, *FXN* transcriptional deficiency initiation is the major cause of frataxin deficiency, and it is related to the extent of repressive chromatin from the GAA expansion [13,30]. GAA expanded repeats can adopt abnormal structures such as the triplex-based “sticky” DNA [31,32] and R-loops [33,34], and result in the epigenetic silencing of a closely linked transgene [35,36]. The way in which the expanded GAA triplet can cause such a decrease in the expression of this protein is still poorly understood, although different findings suggest two non-exclusive hypotheses. The first hypothesis describes the formation of an unusual non-B DNA structure or DNA/RNA hybrids that prevent the advance of RNA polymerase II, based on the experiments of Ohshima and collaborators [37] (reviewed by [4]). The second hypothesis proposes a “heterochromatinization” induced by the GAA-triplet expansion, based on the studies of Saveliev and co-workers [35] (reviewed by [38]). There are different marks of heterochromatin in the vicinity of the expansion, including histone deacetylation (H3K9ac, H3K14ac, and H4K5ac), histone trimethylation (H3K9me3 and H3K27me3), and CpG DNA methylation [39,40,41,42]. These changes have seen observed both upstream and downstream from the GAA expansion, although marks of heterochromatin were more prominent immediately upstream, but still within intron 1 [30].

Most studies on *FXN* gene regulation have been focused on the region around the minimal promoter [14,19] and the region of intron 1 in which GAA expansion causes FRDA [17]. However, numerous studies have shown that both the intronic regions of a gene and the intergenic zones may play important roles in genomic regulation [43]. Due to the increasing evidence suggesting that epigenetic changes could be involved in the reduced levels of *FXN* transcripts [44,45], the aim of the present study was to obtain a more detailed view of the whole genomic *FXN* locus from an epigenetic point of view. Thus, in this work, we used data generated by ENCODE [46] and Roadmap consortia [47] to perform a bioinformatic study through data mining that allowed us to identify new possible regulatory elements in the complete locus of the *FXN* gene.

### 3.1. Study of the FXN Locus in the Cell Lines Provided by the ENCODE Consortium

The objective of the ENCyclopedia Of DNA Elements (ENCODE) consortium is to identify all the functional elements present in the human genome [46,48]. To do this, the consortium has recruited and integrated a large variety of experimental data in a free access portal [49]. The interface developed by the University of California Santa Cruz (https://genome-euro.ucsc.edu) is the main tool to access these data [50,51]. 

As was previously mentioned, chromatin plays an important role in controlling DNA access and specific histone marks are related to promoters, enhancers, transcribed regions, and silenced regions [52,53]. Combinations of these modifications can provide even more precise insight into chromatin state [54]. Within the ENCODE consortium, two research groups have developed different chromatin state annotation algorithms: ChromHMM [55,56] and Segway [57,58]. Although with some differences, both methods share many key features and employ closely related probabilistic models. As ChromHMM offers more cell lines, we focused on this method for the analysis of the *FXN* locus, although none of them have a neural origin [59]: B-lymphoblastoid cells (GM12876), embryonic stem cells (H1-hESC), erythrocytic leukemia cells (K562), hepatocellular carcinoma cells (HepG2), umbilical vein endothelial cells (HUVEC), mammary epithelial cells (HMEC), skeletal muscle myoblasts (HSMM), normal epidermal keratinocytes (NHEK), and normal lung fibroblasts (NHLF). 

The mapping of the different chromatin states was done using a ChIP-seq method with antibodies against the following histone modifications: histone H3 lysine 4 methylation (H3K4me1), a mark predominantly associated with enhancers; H3K4me2, associated with promoters and enhancers; H3K4me3, related to promoters; H3K36me3 and H4K20me1, associated with transcribed regions; H3K27me3, associated with Polycomb-repressed regions; histone 3 lysine 9 acetylation (H3K9ac) and H3K27ac, associated with active regulatory regions; and CTCF, a sequence-specific insulator protein. The chromatin states were established using a multivariate Hidden Markov Model (HMM) that models the combinatorial patterns of observed modifications; the recurrent combinations of these marks define 15 chromatin states as repressed, poised, and active promoters, strong and weak enhancers, putative insulators, transcribed regions, and large-scale repressed and inactive domains [54].

**Figure 2:**
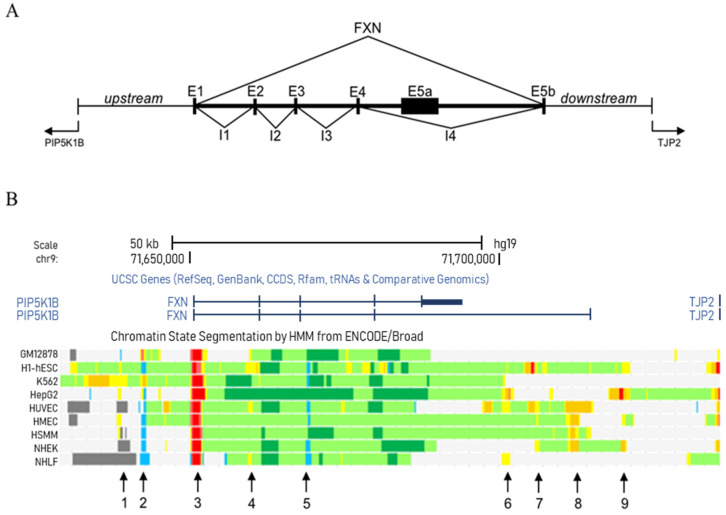
Chromatin states in the *FXN* genomic locus based on ENCODE data. All data were collected from the ENCODE database [46] and visualized in the University of California Santa Cruz browser (https://genome-euro.ucsc.edu). (**A**) Frataxin genomic locus spanning from the 3′end of the upstream gene (PRKACG) to the 5′end of the downstream gene (TJP2) (chromosome 9, 71629000-71736300 region); (**B**) Chromatin states across the *FXN* genomic locus, in which we can highlight nine regions: regions 1 and 2 (within the 5′UTR), region 3 (promoter zone), regions 4–8 (introns) and region 9 (3′UTR). Abbreviations: Ex, Exon x; Ix, Intron x; UTR, Untranslated Region.

The frataxin gene is composed of seven exons, spanning more than 80 kilobases. As we were interested in analyzing not only the entire extension of the frataxin gene but also its upstream and downstream regions, we focused on the chromosomal region between the end of the PRKACG gene, located upstream from the 5′ end of the *FXN* gene, and the beginning of the TJP2 gene, located downstream from the 3′ end (chromosome 9, 71629000–71736300 region) (Figure 2A). In order to determine the most relevant regulatory zones in this genomic region, we only considered those areas in which the epigenetic pattern shown by ENCODE is present in more than 40% of the analyzed cell lines and whose extension exceeds 500 bp.

With these data, the bioinformatic analysis allowed us to identify nine possible regulatory regions within the *FXN* genomic locus (Figure 2B and Table 1): two of them are located in the upstream region of the gene (regions 1 and 2), one in the promoter zone (region 3), five in different introns (regions 4–8) and one in the downstream region of the gene (region 9). Based on these results, the study reveals that intron 4, or more precisely the 3′ end of intron 4 (regions 6–9), might play an important role in gene regulation, since these regions are associated with normal and strong enhancers (Figure 2B and Table 1).

### 3.2. Study on Tissues and Primary Cultures Provided by the Roadmap Consortium

The US National Institutes of Health (NIH) established The Roadmap Epigenomics Program with the goal of elucidating how epigenetic processes contribute to human biology and disease. They provide a publicly accessible resource (http://epigenomegateway.wustl.edu/browser/roadmap) of epigenetic maps in stem cells and ex-vivo tissues, including several human adult brain structures [47]. For the realization of this analysis, different types of tissues were selected, including some of great interest in the study of Friedreich’s Ataxia, such as brain, muscle, pancreatic, and heart tissues. Regarding the epigenetic marks, we focused on five histone modifications, already analyzed in the study carried out with ENCODE data: H3K4me1, H3K4me3, H3K27me3, H3K27ac, and H3K36me3. 

**Figure 3:**
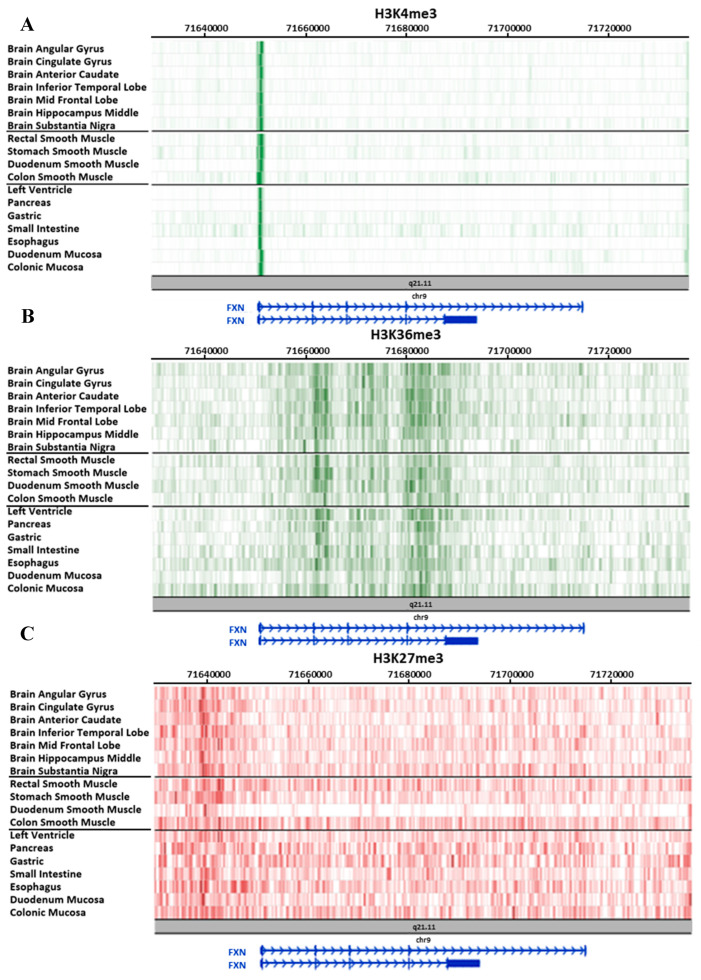
Epigenetic marks with a common pattern across the different tissues provided by Roadmap. All data were collected from the Roadmap database [47] and visualized in its own browser (https://epigenomegateway.wustl.edu/browser/roadmap). (**A**) H3K4me3 mark is related to promoter regions; (**B**) H3K36me3 mark is associated with active transcription; (**C**) H3K27me3 mark is related to heterochromatin. Tissues are organized in three groups: brain, muscle, and other tissues.

On the one hand, the different tissues studied show similar epigenetic patterns for some epigenetic marks. For example, the H3K4me3 mark, associated with promoter zones, coincides in all the compared tissues (Figure 3A), as well as the H3K36me3 mark, associated with active transcribed genes, although the latter shows more intense signals at the initial part of introns 2 and 4, and along intron 3 (Figure 3B). Finally, epigenetic mark H3K27me3, associated with heterochromatin zones, presents an intense signal in the upstream region of the gene, also showing a similar pattern in all analyzed tissues (Figure 3C).

**Figure 4:**
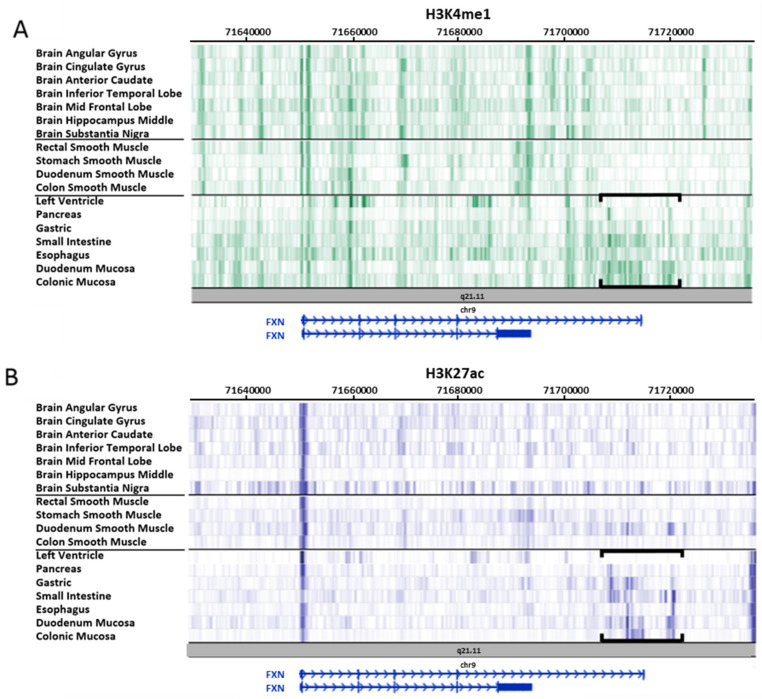
Epigenetic marks of enhancer regions in tissues provided by Roadmap. All data were collected from the Roadmap database [47] and visualized in its own browser (https://epigenomegateway.wustl.edu/browser/roadmap). (**A**) H3K4me1 mark is related to enhancer regions; (**B**) H3K27ac mark is associated with strong enhancers. Tissues are organized in three groups: brain, muscle, and other tissues. Both marks (H3K4me1 and H3K27ac) show tissue-specific patterns, highlighting the region between the 3′end zone of intron 4 and the downstream region closest to the *FXN* gene, where H3K4me1 and H3K27ac signals are almost inexistent in muscle and brain tissues, whereas the remaining tissues present high intensity (region in brackets).

On the other hand, the H3K4me1 mark, related to enhancer regions, shows a more complex epigenomic map, with common patterns among the different groups of tissues analyzed, alongside regions showing specific patterns for each group (Figure 4A). According to the values provided by the Roadmap Consortium, we can differentiate 20 different regions classified as "weak" (signal score below 7.5), "intermediate" (between 7.5 and 10) or "strong" (greater than 10) enhancers (Table 2). In this sense, the region between the end of intron 4 and the downstream region close to *FXN* is of special interest, since in this region the signal of epigenetic mark H3K4me1 is weak in brain and muscle tissues, practically absent in pancreatic and cardiac tissues, and strong in the rest of the analyzed tissues (Figure 4A). Taking into account that brain and muscle tissues are especially affected in FRDA, and that patients with this disease have cardiac muscle atrophies and are prone to developing diabetes, these findings may suggest a direct relationship between the absence of enhancer sequences in this region and their predisposition to be affected in FRDA.

This view is reinforced with the epigenetic map shown by H3K27ac modification, which is an indicator of “strong” enhancers. Similarly, although more clearly, the zone of the *FXN* genomic locus with the most intense signal coincides with the enhancer regions that were previously highlighted (end of intron 4 and downstream region close to *FXN*) and in the same tissue types (Figure 4B).

Finally, we also analyzed the H3K4me1 and H3K27ac marks in the primary cultures provided by the Roadmap Consortium: peripheral blood mononuclear cells, penile foreskin melanocytes and keratinocytes, bone marrow-derived mesenchymal stem cells, and ganglionic eminence-derived neurospheres. In the same way, as already indicated for the different tissues analyzed above, we can clearly distinguish two different patterns in the region near the end of intron 4, where primary cultures of neural origin present a weak signal in both H3K4me1 (enhancers) and H3K27ac (strong enhancers), in contrast to the rest of the cultures, where a strong signal can be observed (data not shown).

## 4. Future Perspectives

Given that over 95% of the human genome reveals no protein-coding information, studies are forced to shift the focus from genomes to epigenomes, with the aim of explaining how the complex diversity of human cells may be caused only by a limited number of genes. Using already published information, we described how different types of human cells and tissues present a different regulatory pattern, including regions inside the frataxin gene and in the upstream and downstream regions of the gene. The region of intron 4 is of particular relevance, due to the differences observed between affected cells and tissues from FRDA patients compared to other tissues that are not affected by the disease. However, it is important to remark that, despite having access to epigenetic studies in brain tissues [47], there are no highly significant data in the most affected tissues in FRDA, such as the cerebellum or the spinal cord.

The findings shown in this study highlight the essential role of certain intronic regions in gene regulation [60,61] and thus support the use of vectors capable of harboring large DNA fragments (whole genomic loci) in gene therapy trials. Vectors encoding FXN cDNA may result in protein overexpression in the heart and cerebellum, as well as in off-target organs, such as the liver, which has been associated with significant cardiotoxicity [62]. Another study of FXN overexpression in cultured human cells also shows that the levels of FXN must be tightly regulated and fine-tuned, with any imbalance leading to oxidative stress and cytotoxicity [63]. In contrast, using Yeast and Bacteria Artificial Chromosomes (YACs and BACs, respectively), it has been shown that large genomic fragments allow tissue-specific expression at physiological levels [64]. Our laboratory has also conducted various studies of FRDA gene therapy using vectors based on HSV-1; this virus was selected for its large storage capacity, which allows the complete packaging of the human frataxin gene. With this approach, we observed a recovery of frataxin levels and a rescue against oxidative stress in fibroblasts of FRDA patients [65]. In addition, it has also been found that the use of a herpesviral vector bearing this BAC allows the long-term expression of FXN in vivo [66]. On the other hand, the different functions of frataxin remain a subject of controversy. A number of different forms of the protein have been described with different specific functions [67,68], and three isoforms of the frataxin protein have also been described [12], with specific cellular locations for each of them. Our laboratory also demonstrated that the BAC vector with the frataxin genomic locus packaged in HSV-1 vectors was able to produce all the different frataxin isoforms described in vitro, both in several neural cultures, including cells derived from the patient, and after injections into mouse cerebellum in vivo [69]. Furthermore, we have also optimized the HSV-1 vector generation process, bringing this technique closer to its wide clinical application [70]. 

Therefore, based on previous evidence and on the results obtained in this bioinformatic study, vectors with complete genomic loci are a promising candidate for gene therapy in FRDA patients, since they contain all the regulatory elements necessary for the physiological expression of frataxin. 

## Figures and Tables

**Table 1:** Putative regulatory regions in the *FXN* genomic locus and associated chromatin state provided by ENCODE.

Chromosome 9			Chromatin State	
Nº	Start	End		
**1**	71638200	71639000		Enhancer
**2**	71642200	71643000		Insulator
**3**	71650400	71652100		Promoter
**4**	71659400	71660200		Enhancer
**5**	71668900	71669600		Insulator
**6**	71700500	71702000		Enhancer
**7**	71704200	71706600		Enhancer
**8**	71710700	71713200		Strong enhancer
**9**	71719600	71721400		Enhancer

**Table 2:** Intensity of the H3K4me1 epigenetic mark across the studied region: “weak” (<7.5; white), “intermediate” (7.5–10; light grey) or “strong” signal (> 10; dark grey). Abbreviations: Down, Downstream region; Ix, Intron x; ORF, Open Reading Frame; Up, Upstream region.

H3K4me1							
		Chromosome 9			Tissues		
		Region	Start	End	Cerebral	Muscular	Others
**Up**		1	71630400	71632000	12.14	13.50	9.67
		2	71634700	71636000	5.71	3.75	10.17
		3	71638300	71639600	8.43	5.50	10.50
		4	71642100	71643100	13.29	9.25	12.17
		5	71650000	71650700	19.71	20.25	14.67
**ORF**	**I1**	6	71651000	71652200	20.14	17.25	16.17
		7	71659300	71660200	12.14	17.25	17.83
	**I2**	8	71661500	71662800	10.43	4.00	4.67
	**I3**	9	71668400	71670400	12.29	11.50	8.67
		10	71678600	71681700	9.71	8.00	9.33
	**I4**	11	71683300	71686300	5.00	4.00	12.67
		12	71690700	71691900	5.29	11.50	5.33
		13	71693000	71694500	16.71	15.50	12.67
		14	71697600	71698700	12.50	6.50	9.67
		15	71700300	71702400	11.86	8.50	15.50
		16	71703700	71705900	8.71	6.50	13.00
		17	71708600	71709200	3.86	2.50	21.33
		18	71713500	71715400	5.57	4.00	13.17
**Down**		19	71718800	71721000	4.71	4.00	14.00
		20	71726500	71727400	12.00	2.25	6.67

## References

[1] Schulz J.B., Boesch S., Bürk K., Durr A., Giunti P., Mariotti C., Pousset F., Schöls L., Vankan P., Pandolfo M. (2009). Diagnosis and treatment of Friedreich ataxia: A European perspective. Nat. Rev. Neurol..

[2] Pandolfo M. (2002). The molecular basis of Friedreich ataxia. Adv. Exp. Med. Biol..

[3] Bürk K. (2017). Friedreich Ataxia: Current status and future prospects. Cerebellum Ataxias.

[4] Cook A., Giunti P. (2017). Friedreich’s ataxia: Clinical features, pathogenesis and management. Br. Med Bull..

[5] Campuzano V., Montermini L., Moltò M.D., Pianese L., Cossée M., Cavalcanti F., Monros E., Rodius F., Duclos F., Monticelli A. (1996). Friedreich’s ataxia: Autosomal recessive disease caused by an intronic GAA triplet repeat expansion. Science.

[6] Al-Mahdawi S., Ging H., Bayot A., Cavalcanti F.B., La Cognata V., Cavallaro S., Giunti P., Pook M.A. (2018). Large Interruptions of GAA Repeat Expansion Mutations in Friedreich Ataxia Are Very Rare. Front. Cell. Neurosci..

[7] Pastore A., Puccio H. (2013). Frataxin: A protein in search for a function. J. Neurochem..

[8] Clark E., Butler J.S., Isaacs C.J., Napierala M., Lynch D.R. (2017). Selected missense mutations impair frataxin processing in Friedreich ataxia. Ann. Clin. Transl. Neurol..

[9] Navarro J.A., Llorens J.V., Soriano S., Botella J.A., Schneuwly S., Martínez-Sebastián M.J., Moltó M.D. (2011). Overexpression of Human and Fly Frataxins in Drosophila Provokes Deleterious Effects at Biochemical, Physiological and Developmental Levels. PLoS ONE.

[10] Li K., Besse E.K., Ha D., Kovtunovych G., Rouault T.A. (2008). Iron-dependent regulation of frataxin expression: Implications for treatment of Friedreich ataxia. Hum. Mol. Genet..

[11] Pianese L., Tammaro A., Turano M., De Biase I., Monticelli A., Cocozza S. (2002). Identification of a novel transcript of X25, the human gene involved in Friedreich ataxia. Neurosci. Lett..

[12] Xia H., Cao Y., Dai X., Marelja Z., Zhou D., Mo R., Al-Mahdawi S., Pook M.A., Leimkühler S., Rouault T.A. (2012). Novel Frataxin Isoforms May Contribute to the Pathological Mechanism of Friedreich Ataxia. PLoS ONE.

[13] Kumari D., Biacsi R.E., Usdin K. (2010). Repeat Expansion Affects Both Transcription Initiation and Elongation in Friedreich Ataxia Cells. J. Biol. Chem..

[14] Greene E., Entezam A., Kumari D., Usdin K. (2005). Ancient repeated DNA elements and the regulation of the human frataxin promoter. Genomics.

[15] Ågren J.A., Clark A.G. (2018). Selfish genetic elements. PLoS Genet..

[16] Nishibuchi G., Déjardin J. (2017). The molecular basis of the organization of repetitive DNA-containing constitutive heterochromatin in mammals. Chromosom. Res..

[17] Greene E., Mahishi L., Entezam A., Kumari D., Usdin K. (2007). Repeat-induced epigenetic changes in intron 1 of the frataxin gene and its consequences in Friedreich ataxia. Nucleic Acids Res..

[18] Li K., Singh A., Crooks D.R., Dai X., Cong Z., Pan L., Ha D., Rouault T.A. (2010). Expression of Human Frataxin Is Regulated by Transcription Factors SRF and TFAP2. PLoS ONE.

[19] De Biase I., Chutake Y.K., Rindler P.M., Bidichandani S.I. (2009). Epigenetic Silencing in Friedreich Ataxia Is Associated with Depletion of CTCF (CCCTC-Binding Factor) and Antisense Transcription. PLoS ONE.

[20] Bushey A.M., Dorman E.R., Corces V.G. (2008). Chromatin Insulators: Regulatory Mechanisms and Epigenetic Inheritance. Mol. Cell.

[21] Shimizu R., Lan N.N., Tai T.T., Adachi Y., Kawazoe A., Mu A., Taketani S. (2014). p53 directly regulates the transcription of the human frataxin gene and its lack of regulation in tumor cells decreases the utilization of mitochondrial iron. Gene.

[22] Sahdeo S., Scott B.D., McMackin M.Z., Jasoliya M., Brown B., Wulff H., Perlman S.L., Pook M.A., Cortopassi G.A. (2014). Dyclonine rescues frataxin deficiency in animal models and buccal cells of patients with Friedreich’s ataxia. Hum. Mol. Genet..

[23] Yang B., Fritsche K.L., Beversdorf D.Q., Gu Z., Lee J.C., Folk W.R., Greenlief C.M., Sun G.Y. (2019). Yin-Yang Mechanisms Regulating Lipid Peroxidation of Docosahexaenoic Acid and Arachidonic Acid in the Central Nervous System. Front. Neurol..

[24] Yang B., Li R., Greenlief C.M., Fritsche K., Gu Z., Cui J., Lee J.C., Beversdorf D., Sun G.Y. (2018). Unveiling anti-oxidative and anti-inflammatory effects of docosahexaenoic acid and its lipid peroxidation product on lipopolysaccharide-stimulated BV-2 microglial cells. J. Neuroinflamm..

[25] Inbar-Feigenberg M., Choufani S., Butcher D.T., Roifman M., Weksberg R. (2013). Basic concepts of epigenetics. Fertil. Steril..

[26] Molina-Serrano D., Schiza V., Kirmizis A. (2013). Cross-talk among epigenetic modifications: Lessons from histone arginine methylation. Biochem. Soc. Trans..

[27] Luger K., Mäder A.W., Richmond R.K., Sargent D.F., Richmond T.J. (1997). Crystal structure of the nucleosome core particle at 2.8 Å resolution. Nature.

[28] Murr R. (2010). Interplay between different epigenetic modifications and mechanisms. Adv. Genet..

[29] Musselman C.A., LaLonde M.-E., Côté J., Kutateladze T. (2012). Perceiving the epigenetic landscape through histone readers. Nat. Struct. Mol. Biol..

[30] Chutake Y.K., Costello W., Lam C., Bidichandani S.I. (2014). Altered nucleosome positioning at the transcription start site and deficient transcriptional initiation in Friedreich ataxia. J. Biol. Chem..

[31] Rajeswari M.R. (2012). DNA triplex structures in neurodegenerative disorder, Friedreich’s ataxia. J. Biosci..

[32] Sakamoto N., Chastain P.D., Parniewski P., Ohshima K., Pandolfo M., Griffith J.D., Wells R.D. (1999). Sticky DNA: Self-association properties of long GAA.TTC repeats in R.R.Y triplex structures from Friedreich’s ataxia. Mol. Cell.

[33] Grabczyk E., Mancuso M., Sammarco M. (2007). A persistent RNA.DNA hybrid formed by transcription of the Friedreich ataxia triplet repeat in live bacteria, and by T7 RNAP in vitro. Nucleic Acids Res..

[34] Groh M., Lufino M.M.P., Wade-Martins R., Gromak N. (2014). R-loops Associated with Triplet Repeat Expansions Promote Gene Silencing in Friedreich Ataxia and Fragile X Syndrome. PLoS Genet..

[35] Saveliev A., Everett C., Sharpe T., Webster Z., Festenstein R. (2003). DNA triplet repeats mediate heterochromatin-protein-1-sensitive variegated gene silencing. Nature.

[36] Wells R.D. (2008). DNA triplexes and Friedreich ataxia. FASEB J..

[37] Ohshima K., Montermini L., Wells R.D., Pandolfo M. (1998). Inhibitory Effects of Expanded GAA·TTC Triplet Repeats from Intron I of the Friedreich Ataxia Gene on Transcription and Replicationin Vivo. J. Biol. Chem..

[38] Marmolino D., Acquaviva F. (2009). Friedreich’s Ataxia: From the (GAA) n Repeat Mediated Silencing to New Promising Molecules for Therapy. Cerebellum.

[39] Punga T., Bühler M. (2010). Long intronic GAA repeats causing Friedreich ataxia impede transcription elongation. EMBO Mol. Med..

[40] Evans-Galea M.V., Carrodus N., Rowley S., Corben L.A., Tai G., Saffery R., Galati J.C., Wong N.C., Craig J.M., Lynch D.R. (2012). FXN methylation predicts expression and clinical outcome in Friedreich ataxia. Ann. Neurol..

[41] Castaldo I., Pinelli M., Monticelli A., Acquaviva F., Giacchetti M., Filla A., Sacchetti S., Keller S., Avvedimento V.E., Chiariotti L. (2008). DNA methylation in intron 1 of the frataxin gene is related to GAA repeat length and age of onset in Friedreich ataxia patients. J. Med Genet..

[42] Al-Mahdawi S., Pinto R.M., Ismail O., Varshney D., Lymperi S., Sandi C., Trabzuni D., Pook M.A. (2007). The Friedreich ataxia GAA repeat expansion mutation induces comparable epigenetic changes in human and transgenic mouse brain and heart tissues. Hum. Mol. Genet..

[43] Rivera C.M., Ren B. (2013). Mapping human epigenomes. Cell.

[44] Nageshwaran S., Festenstein R. (2015). Epigenetics and Triplet-Repeat Neurological Diseases. Front. Neurol..

[45] Sandi C., Sandi M., Virmouni S.A., Al-Mahdawi S., Pook M.A. (2014). Epigenetic-based therapies for Friedreich ataxia. Front. Genet..

[46] Consortium E.P. (2012). An integrated encyclopedia of DNA elements in the human genome. Nature.

[47] Kundaje A., Meuleman W., Ernst J., Bilenky M., Yen A., Heravi-Moussavi A., Kheradpour P., Zhang Z., Wang J., Roadmap Epigenomics Consortium (2015). Integrative analysis of 111 reference human epigenomes. Nature.

[48] (2004). The ENCODE Project Consortium The ENCODE (ENCyclopedia Of DNA Elements) Project. Science.

[49] Luo Y., Hitz B.C., Gabdank I., A Hilton J., Kagda M.S., Lam B., Myers Z., Sud P., Jou J., Lin K. (2020). New developments on the Encyclopedia of DNA Elements (ENCODE) data portal. Nucleic Acids Res..

[50] Thomas D.J., Rosenbloom K.R., Clawson H., Hinrichs A.S., Trumbower H., Raney B.J., Karolchik N., Barber G.P., Harte R.A., Hillman-Jackson J. (2006). The ENCODE Project at UC Santa Cruz. Nucleic Acids Res..

[51] Sloan C.A., Chan E., Davidson J., Malladi V., Strattan J.S., Hitz B.C., Gabdank I., Narayanan A., Ho M., Ho M.C. (2015). ENCODE data at the ENCODE portal. Nucleic Acids Res..

[52] Geiman T.M., Robertson K.D. (2002). Chromatin remodeling, histone modifications, and DNA methylation?how does it all fit together?. J. Cell. Biochem..

[53] Cavalli G., Heard E. (2019). Advances in epigenetics link genetics to the environment and disease. Nature.

[54] Ernst J., Kellis M. (2010). Discovery and characterization of chromatin states for systematic annotation of the human genome. Nat. Biotechnol..

[55] Ernst J., Kellis M. (2012). ChromHMM: Automating chromatin-state discovery and characterization. Nat. Methods.

[56] Ernst J., Kellis M. (2017). Chromatin-state discovery and genome annotation with ChromHMM. Nat. Protoc..

[57] Chan R.C., Libbrecht M.W., Roberts E.G., Bilmes J.A., Noble W.S., Hoffman M.M. (2017). Segway 2.0: Gaussian mixture models and minibatch training. Bioinformatics.

[58] Hoffman M.M., Ernst J., Wilder S., Kundaje A., Harris R.S., Libbrecht M., Giardine B., Ellenbogen P.M., Bilmes J.A., Birney E. (2012). Integrative annotation of chromatin elements from ENCODE data. Nucleic Acids Res..

[59] Ernst J., Kheradpour P., Mikkelsen T.S., Shoresh N., Ward L.D., Epstein C.B., Zhang X., Wang L., Issner R., Coyne M. (2011). Mapping and analysis of chromatin state dynamics in nine human cell types. Nature.

[60] Osman E., Miller M.R., Robbins K.L., Lombardi A.M., Atkinson A.K., Brehm A.J., Lorson C.L. (2014). Morpholino antisense oligonucleotides targeting intronic repressor Element1 improve phenotype in SMA mouse models. Hum. Mol. Genet..

[61] Ott C.J., Blackledge N.P., Kerschner J.L., Leir S.-H., Crawford G.E., Cotton C.U., Harris A. (2009). Intronic enhancers coordinate epithelial-specific looping of the active CFTR locus. Proc. Natl. Acad. Sci. USA.

[62] Belbellaa B., Reutenauer L., Messaddeq N., Monassier L., Puccio H. (2020). High levels of frataxin overexpression leads to mitochondrial and cardiac toxicity in mouse models. BioRxiv.

[63] Vannocci T., Manzano R.N., Beccalli O., Bettegazzi B., Grohovaz F., Cinque G., De Riso A., Quaroni L., Codazzi F., Pastore A. (2018). Adding a temporal dimension to the study of Friedreich’s ataxia: The effect of frataxin overexpression in a human cell model. Dis. Model. Mech..

[64] Taylor T.N., Potgieter D., Anwar S., Senior S.L., Janezic S., Threlfell S., Ryan B., Parkkinen L., Deltheil T., Cioroch M. (2013). Region-specific deficits in dopamine, but not norepinephrine, signaling in a novel A30P α-synuclein BAC transgenic mouse. Neurobiol. Dis..

[65] Gomez-Sebastian S., Gimenez-Cassina A., Diaz-Nido J., Lim F., Wade-Martins R. (2007). Infectious delivery and expression of a 135 kb human FRDA genomic DNA locus complements Friedreich’s ataxia deficiency in human cells. Mol. Ther..

[66] Giménez-Cassina A., Wade-Martins R., Gómez-Sebastián S., Corona J.C., Lim F., Diaz-Nido J. (2011). Infectious delivery and long-term persistence of transgene expression in the brain by a 135-kb iBAC-FXN genomic DNA expression vector. Gene Ther..

[67] Gakh O., Bedekovics T., Duncan S.F., Smith D.Y., Berkholz N.S., Isaya G. (2010). Normal and Friedreich Ataxia Cells Express Different Isoforms of Frataxin with Complementary Roles in Iron-Sulfur Cluster Assembly*. J. Biol. Chem..

[68] Schmucker S., Argentini M., Carelle-Calmels N., Martelli A., Puccio H. (2008). The in vivo mitochondrial two-step maturation of human frataxin. Hum. Mol. Genet..

[69] Perez-Luz S., Giménez-Cassina A., Fernández-Frías I., Wade-Martins R., Diaz-Nido J. (2015). Delivery of the 135 kb human frataxin genomic DNA locus gives rise to different frataxin isoforms. Genomics.

[70] Fernández-Frías I., Pérez-Luz S., Díaz-Nido J. (2020). Enhanced Production of Herpes Simplex Virus 1 Amplicon Vectors by Gene Modification and Optimization of Packaging Cell Growth Medium. Mol. Ther. Methods Clin. Dev..

